# Cutaneous, Intranasal Blastomycosis Infection in Two Patients from Southern West Virginia: Diagnostic Dilemma

**DOI:** 10.7759/cureus.2095

**Published:** 2018-01-21

**Authors:** Aaron R Kuzel, Muhammad Uzair Lodhi, Intekhab Askari Syed, Tehmina Zafar, Umar Rahim, Mehenaz Hanbazazh, Nadia Naumova, Mustafa Rahim

**Affiliations:** 1 Department of Emergency Medicine, Lincoln Memorial University-Debusk College of Osteopathic Medicine; 2 Medical Student, Department of Medicine, Raleigh General Hospital, Beckley, Wv; 3 Pre-Medical Student, Department of Sciences, Queens University of Charlotte, Nc; 4 Pathology Resident, West Virginia University School of Medicine; 5 Pathologist, West Virginia University, Wv; 6 Assistant Clinical Professor of Internal Medicine, West Virginia University School of Medicine

**Keywords:** blastomycosis, blastomyces dermatitidis, beckley, west virginia, cutaneous blastomycosis, intranasal blastomycosis, dimorphic fungus, squamous cell carcinoma, intranasal squamous mucosa biopsy, blastomyces gilchristii

## Abstract

Blastomycosis is a dimorphic fungus caused by the species of Blastomyces dermatitidisand Blastomyces gilchristii, which are endemic to the Ohio River and Mississippi River Valleys. It is commonly found in soil or decomposing wood. It is capable of infecting both immunocompromised and immunocompetent patients via the respiratory tract by inhaling conidia, where it may remain asymptomatic for a prolonged period of time. Extrapulmonary complications can occur in disseminated disease due to haematogenous spread from the lungs to other organ systems. Haematogenous dissemination from the lungs occurs most commonly to the skin. Although rare, primary cutaneous blastomycosis can also occur with direct inoculation through the trauma of the skin. Patients presenting with cutaneous blastomycosis are often misdiagnosed with malignant neoplasms and may not be appropriately managed until further testing and tissue biopsy. Currently, there are only four previous case reports of blastomycosis presenting in the intranasal region. We report two cases of cutaneous blastomycosis of the nasal passages and upper lip with pulmonary manifestations in Southern West Virginia. These patients first presented with cutaneous symptoms, which were originally treated for melanoma and squamous cell carcinoma and were later diagnosed with systemic blastomycosis*.*

## Introduction

Blastomycosis is a dimorphic fungus caused by the species of Blastomyces dermatitidisand Blastomyces gilchristii*,* that grow as a filamentous mold in temperatures less than 37°C, and as yeast in animal host organisms above 37°C. Blastomycosis is endemic in the soil of Ohio and Mississippi River Valleys as well as the region of the Great Lakes and southeastern states of the United States (US) [1-3]. The incidence of blastomycosis in these regions is very uncommon with approximately one case per 100,000 population in states, including Arkansas, Illinois, Kentucky, Louisiana, Mississippi, North Carolina, Tennessee, and Wisconsin [4]. Blastomycosis can infect both immunocompetent as well as immunocompromised patients and manifests as a pulmonary disease due to airborne spores being inhaled. Once the spores are inhaled, they are transformed into yeast and are then able to evade the host’s immune system by producing a thick wall that is impenetrable to phagocytosis by macrophages [4]. Blastomycosis commonly manifests as acute pneumonia or a chronic pulmonary infection, which may remain asymptomatic or dormant for prolonged periods of time. A severe pulmonary complication seen with blastomycosis infection is acute respiratory distress syndrome (ARDS), which can prove deadly as it is difficult to detect the disease prior to symptomatic presentation. Extrapulmonary conditions occur approximately in 25-30% of patients due to haematogenous dissemination from the lungs to other organ systems. Haematogenous dissemination from the pulmonary system occurs most commonly to the skin [5]. Although rare, primary cutaneousblastomycosis can also occur with direct inoculation through a trauma of the skin [4, 6].

Initially,blastomycosis may present with flu-like symptoms, but as mentioned previously, the majority of cases are asymptomatic. Cutaneous blastomycosis usually appears as vegetative plaques that often have ulcerations with possible accompanying lymphadenopathy. Other clinical findings may include epididymitis, prostatitis, orchitis, and occasionally neurological conditions, such as meningitis or abscesses. Often, the cases of cutaneous blastomycosis are mistaken for malignant neoplasms and hence may not be discovered until further testing and biopsy. Blastomycosis has been mistaken for keratoacanthoma, cutaneous lesions seen in tertiary syphilis, cutaneous tuberculosis, and most commonly, squamous cell carcinoma [7]. At the present time, there are only four previous case reports of blastomycosis presenting in the intranasal region [8]. Presentation of blastomycosis of the head and neck is most commonly seen in the larynx, but this presentation in the intranasal region is very rare [9].

## Case presentation

Case 1

**Figure 1:**
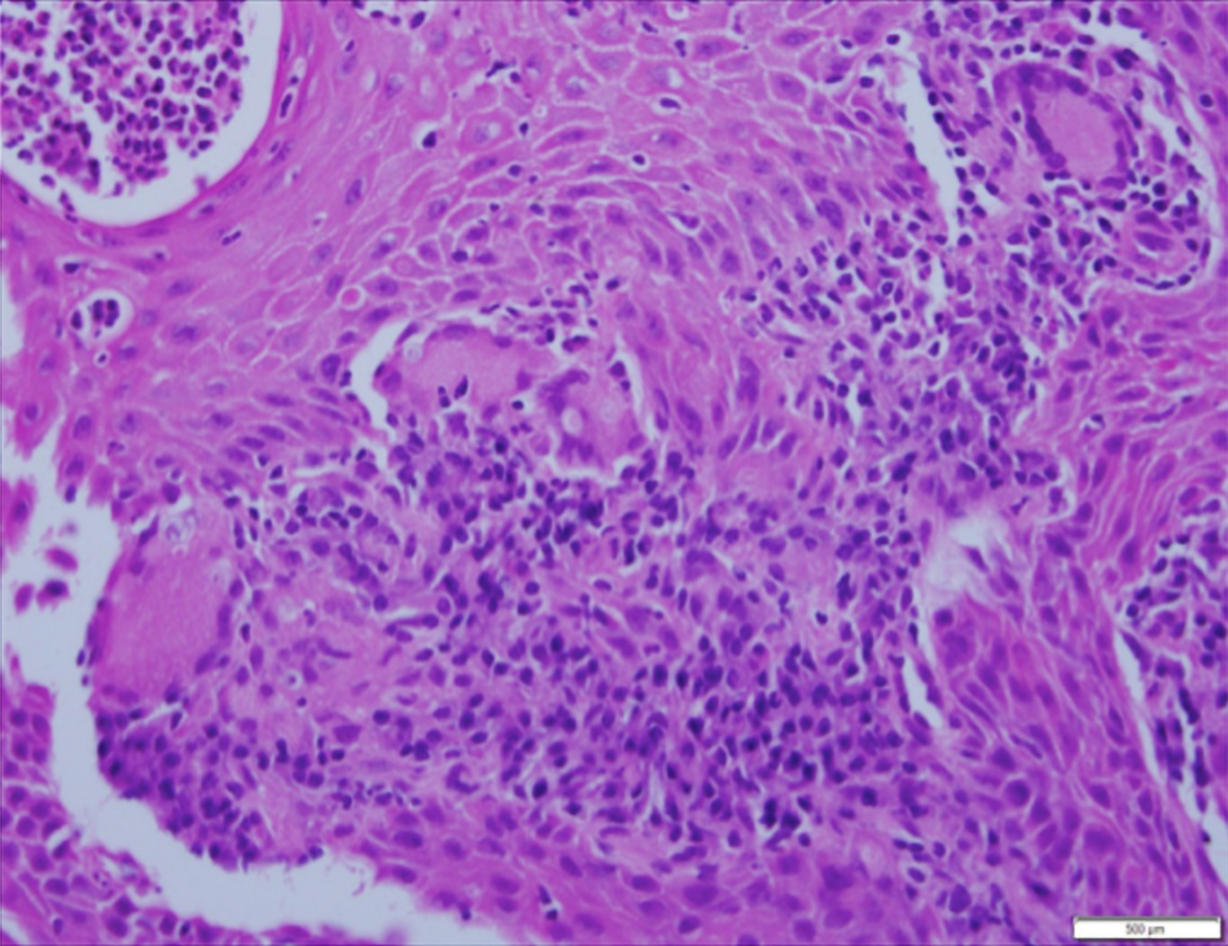
Biopsy of the intranasal squamous mucosa (Hematoxylin and eosin stain) showing acute inflammation and multiple giant cells

**Figure 2:**
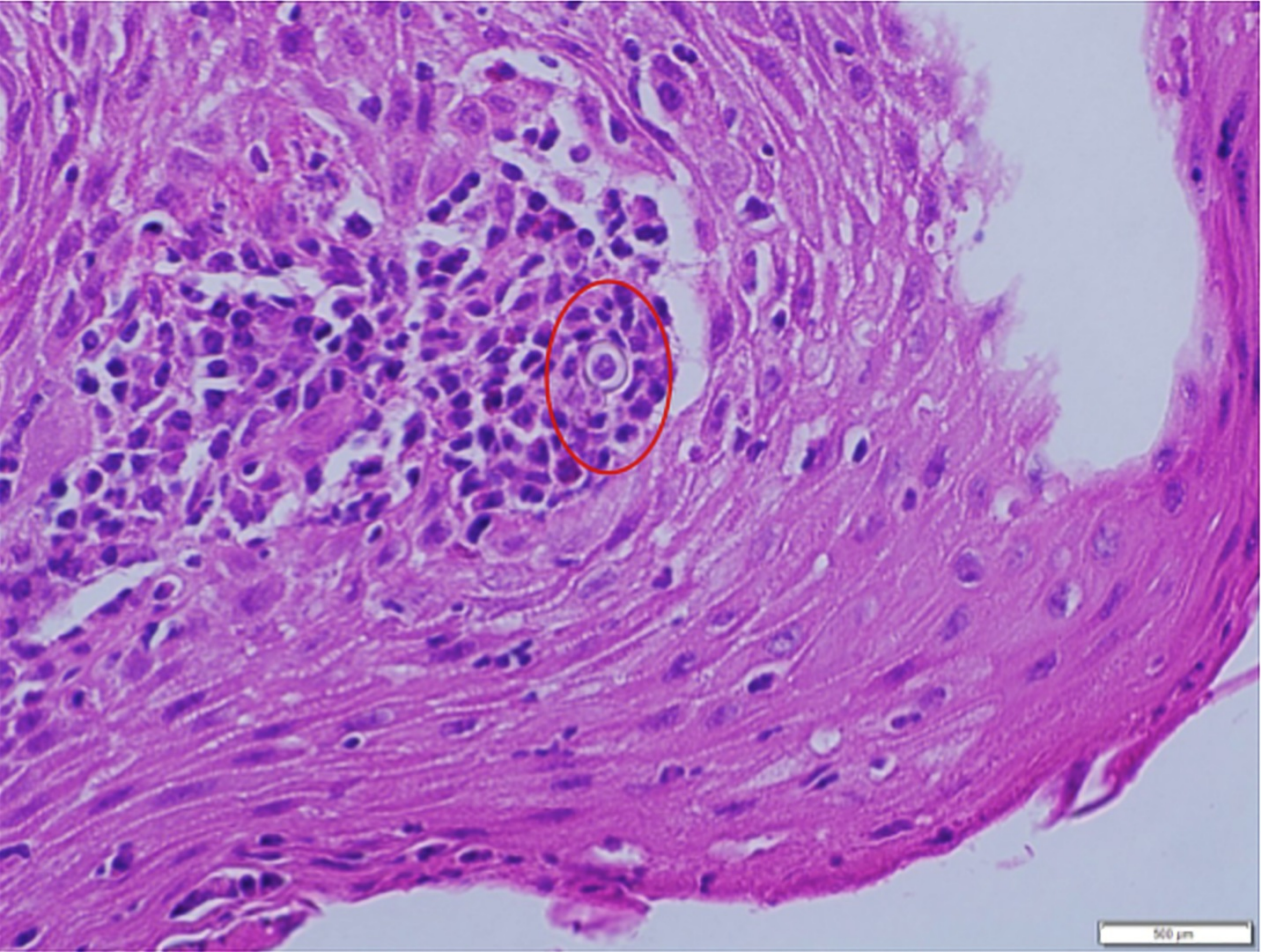
Yeast-like organisms with thick refractile wall and broad-based budding (red circle)

**Figure 3:**
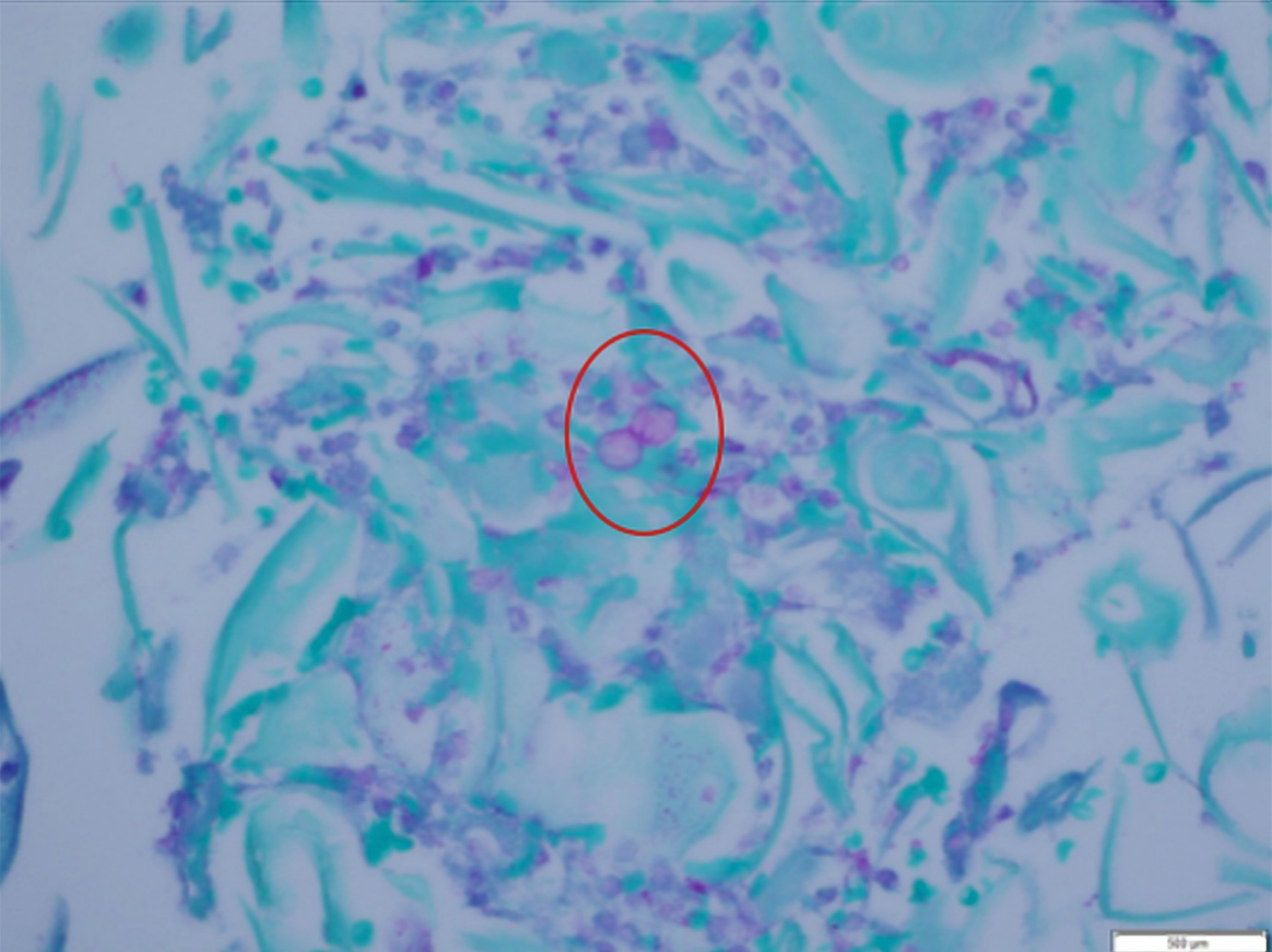
Periodic acid-Schiff (PAS) stain showing blastomycosis with characteristic broad-based budding (red circle)

**Figure 4:**
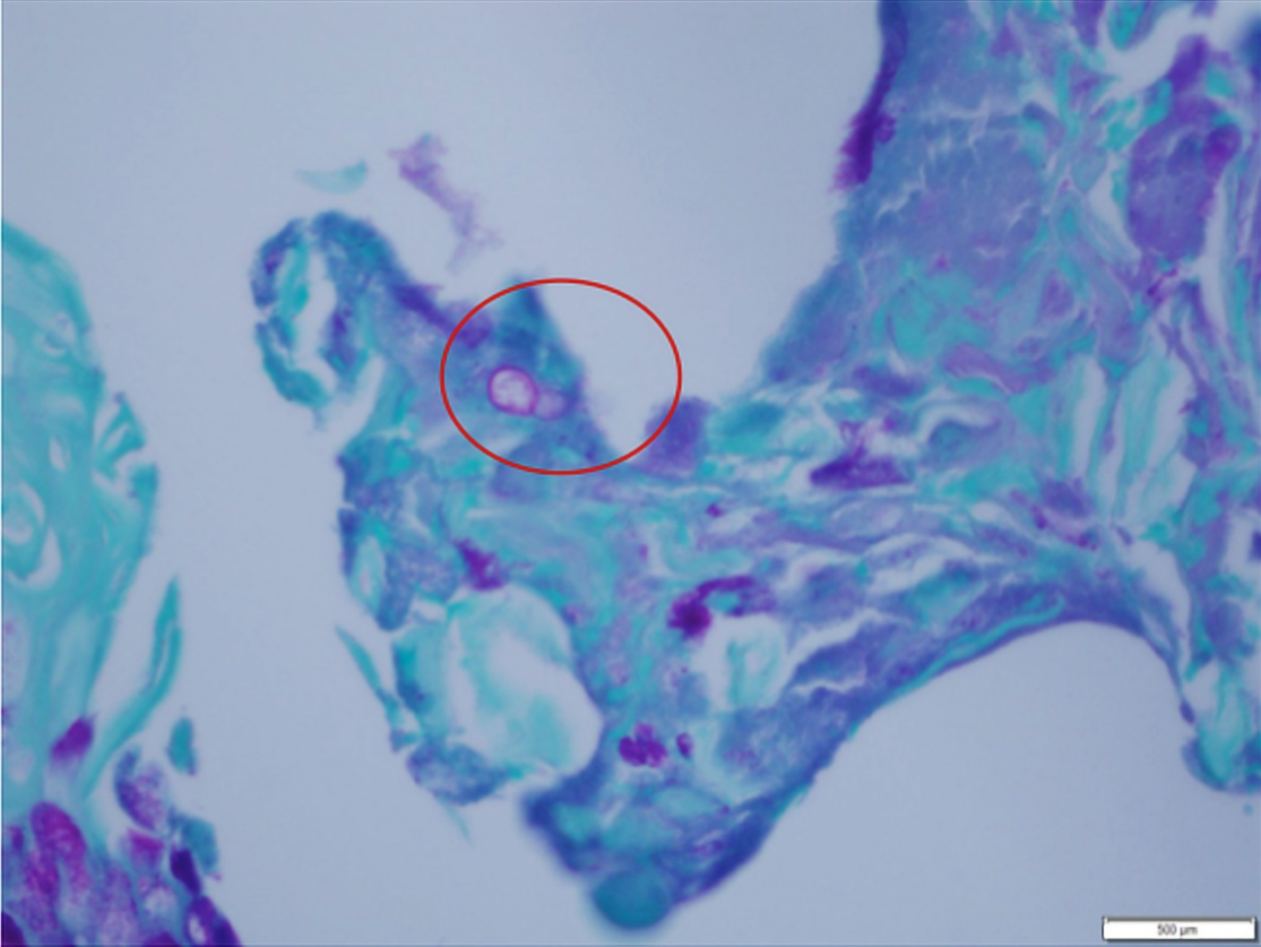
Periodic acid-Schiff (PAS) stain showing one cell in particular and the large based budding as daughter cell is being produced (red circle)

**Figure 5:**
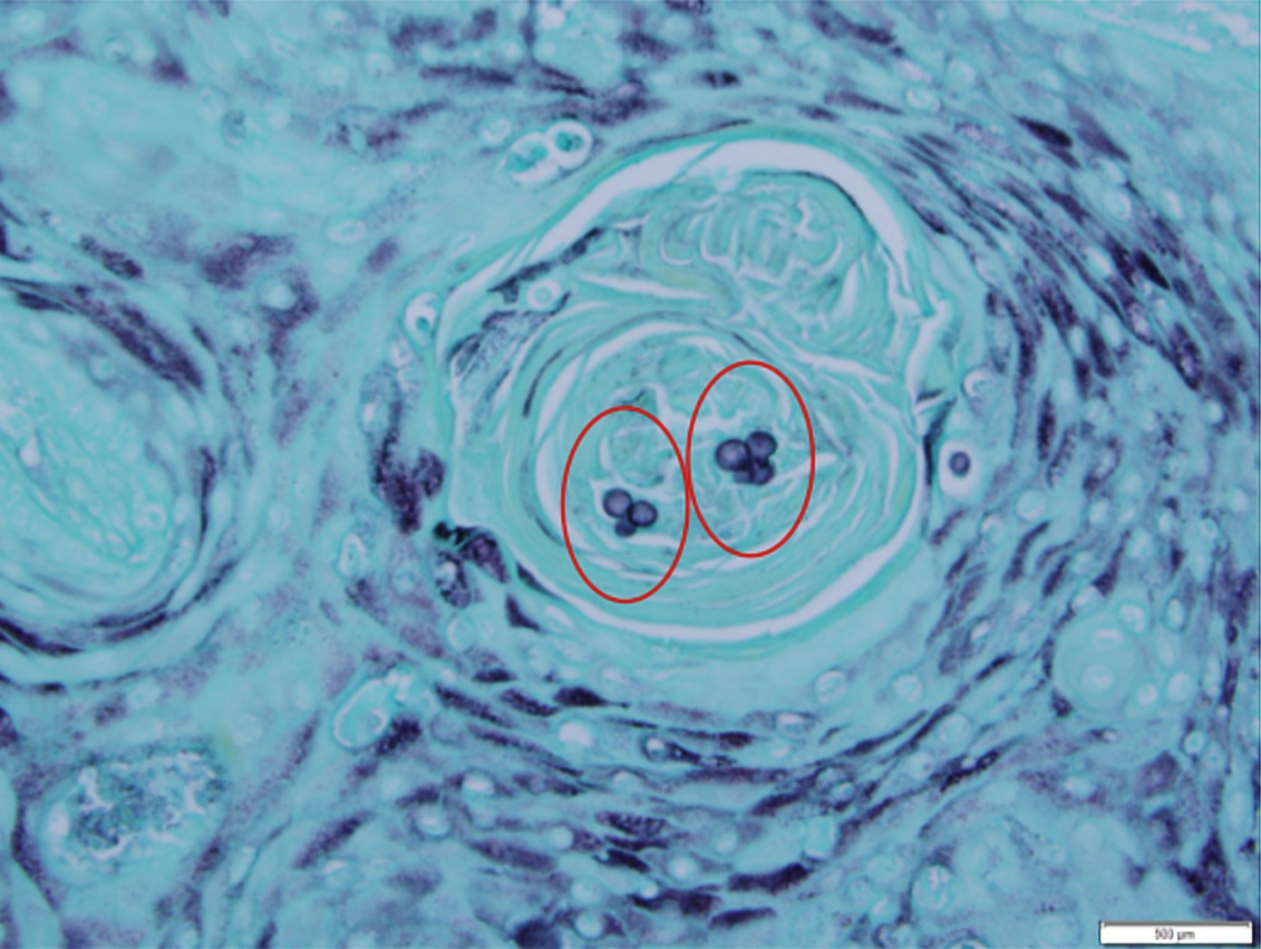
Grocott's methenamine silver (GMS) stain showing budding cells of blastomycosis (red circles)

**Figure 6:**
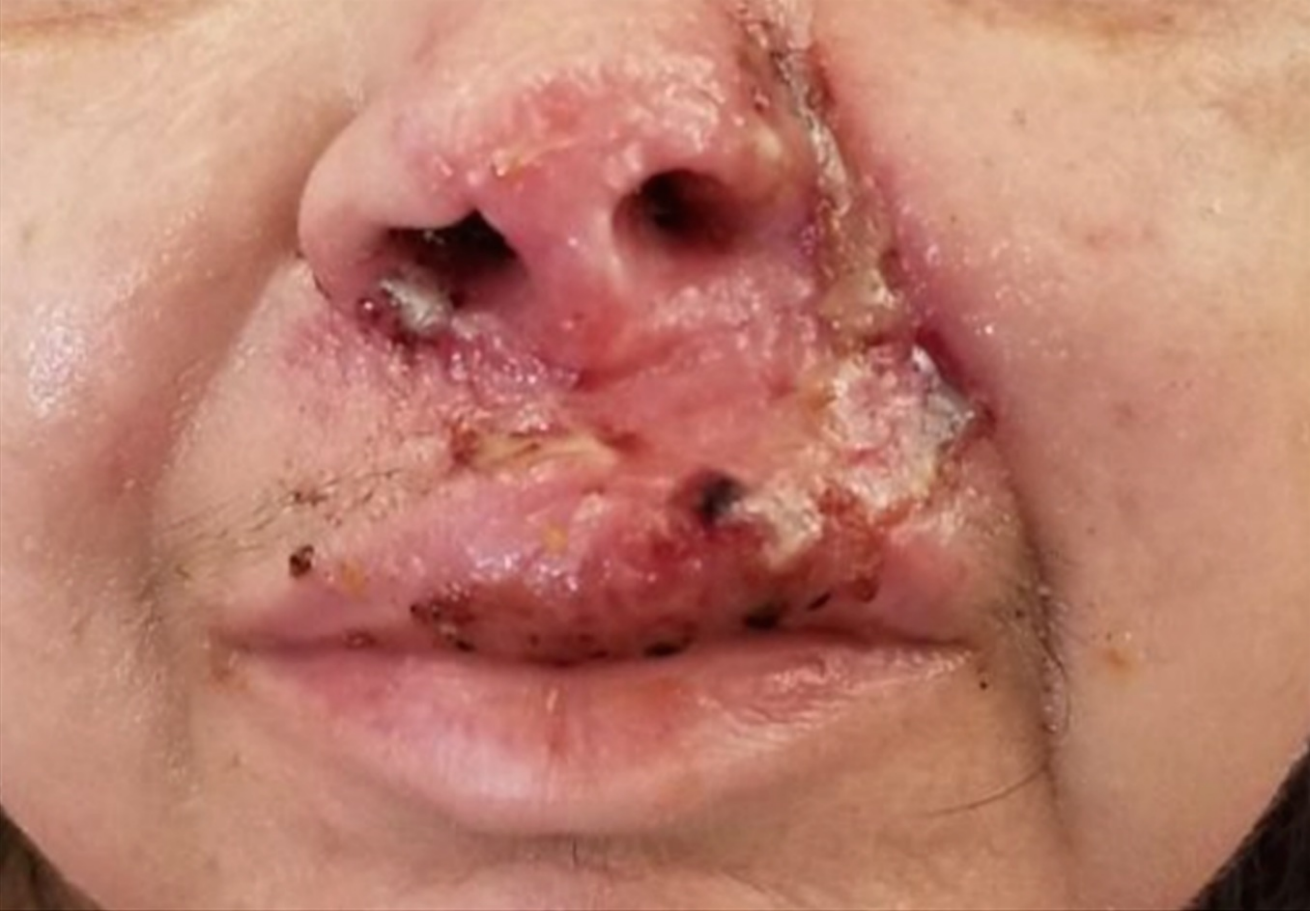
Post-treatment (after itraconazole and debridement of fungating nasal/upper lip lesion) picture showing cutaneous disseminated blastomycosis on nose

The first case of intranasal, cutaneous blastomycosis occurred in a 55-year-old female from Southern West Virginia. She first noticed a small scab-like area on her nose eight months prior to initial presentation to the hospital. The patient had also been diagnosed with a “fungal pulmonary infection” but did not seek treatment for this condition. She was brought to medical attention for the nasal mass due to a maggot infestation and ulceration. A relative of the patient stated that the mass had enlarged progressively since the mass first appeared (in the span of months) and that the patient loved the outdoors but had not traveled outside West Virginia in at least 10 years. At presentation, the mass had enveloped the left nasal passageway and progressed to involve the left upper lip of the patient with an ulcerative lesion and a maggot infestation. She did not complain of pain or discomfort, no difficulty breathing, drinking, eating, or speaking. No fever or other lesions were noted. She had a past medical history of diabetes and arthritis of which she was non-compliant with medications. Her primary care physician began to treat the patient for the possibility of “Aspergillosis” for the pulmonary complications and assessed the nasal mass for melanoma. She was transferred to a larger medical center where she received further testing and evaluation. A computed tomography (CT) scan showed an ulcerated nasal mass and underlying sclerotic bone. The nasal tissue biopsy showed squamous epithelium with florid pseudoepitheliomatous hyperplasia and suppurative granulomatous inflammation, containing numerous multinucleated giant cells with fungal organisms. The organism was characterized by a thick refractile wall and broad-based budding. This can be seen with the hematoxylin and eosin stain (H&E) (Figures 1-2) and best highlighted by periodic acid-Schiff (PAS) (Figures 3-4) and Grocott's methenamine silver (GMS) stains (Figure 5). Cultures for anaerobic bacteria, fungus, and acid-fast bacilli (AFB) were obtained. Blastomycosis antigen tested positive. Chest x-ray was unremarkable. The patient was provided a 14-day course of ivermectin to neutralize the maggots. In the hospital, the patient underwent debulking and debridement of the facial region and was placed on a six to 12-month course of anti-fungal treatment with itraconazole. Follow-up with the patient two months after debridement and itraconazole treatment showed a decrease in the size of the mass and resolution of her pulmonary symptoms. Unfortunately, the pre-treatment gross pictures of intranasal blastomycosis are not available. Her post-treatment picture at two months is shown below (Figure 6). 

 

Case  2

**Figure 7:**
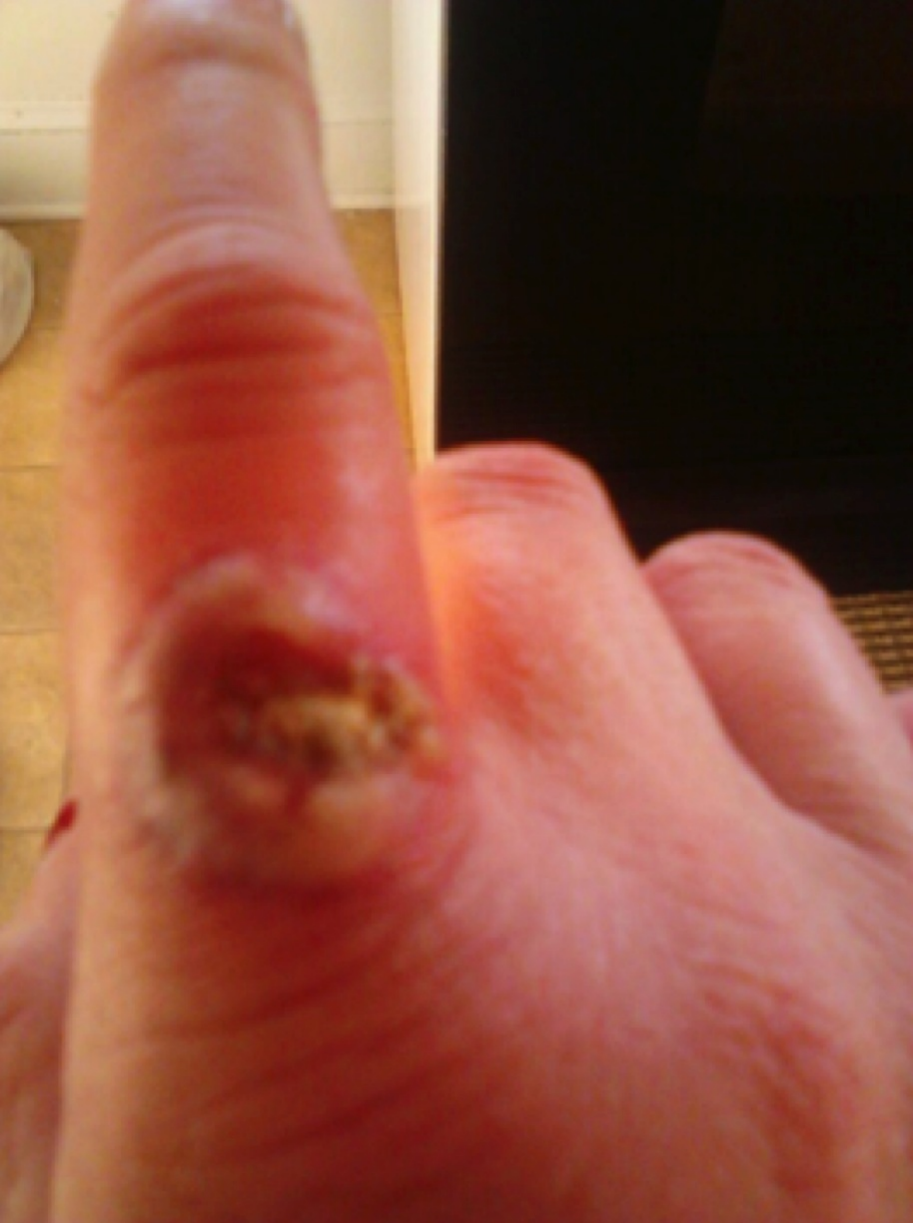
Ulcerating blastomycosis lesion on the right index finger, observed during physical exam

**Figure 8:**
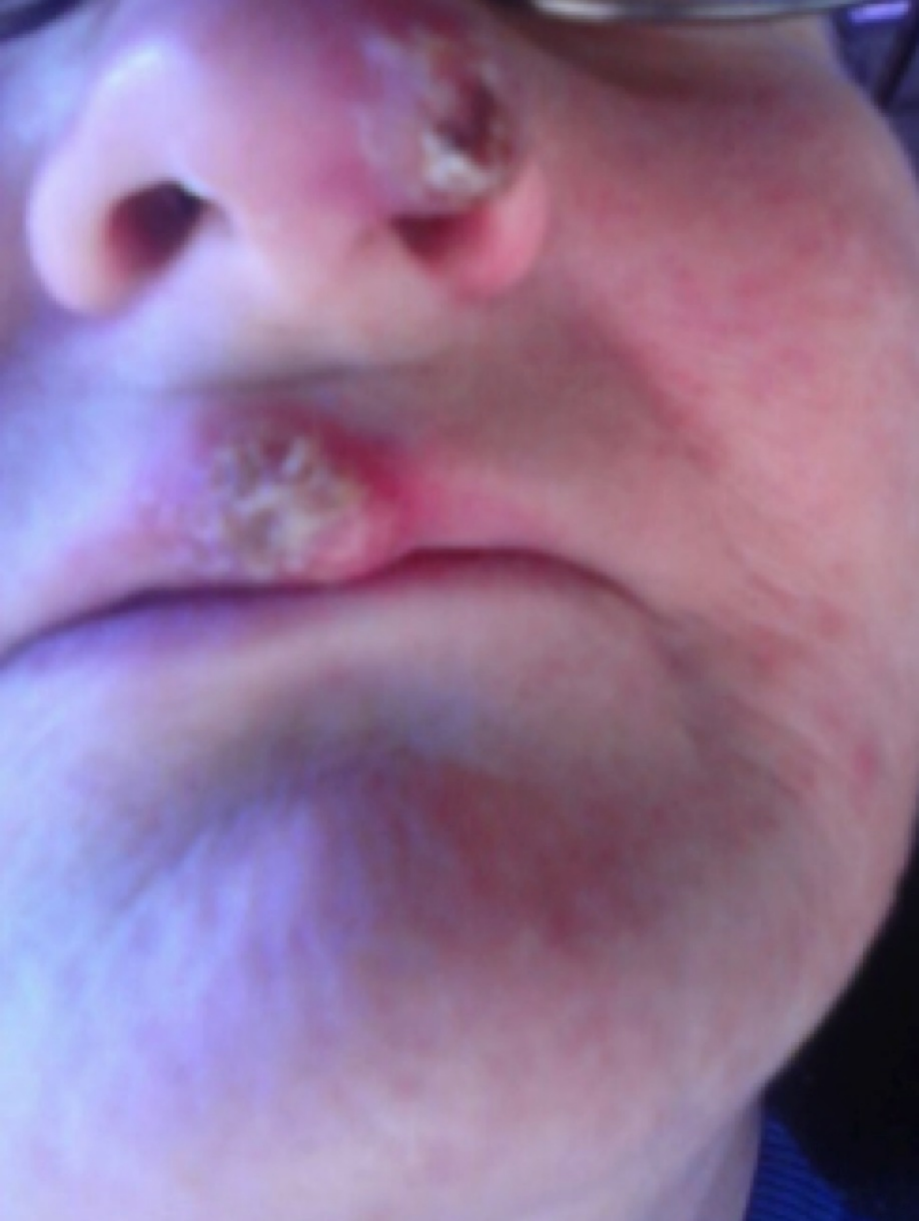
Blastomycosis lesion on left intranasal passageway and middle of upper lip, observed during physical examination

The second case of intranasal, cutaneous blastomycosis occurred in a 49-year-old female, also from Southern West Virginia. The patient first presented with symptoms of wheezing and cough, which she attributed to a progressive course of the “flu.” She had a medical history of diabetes and had been compliant with medication. She was previously a smoker and denied any recent travel. She stated that these pulmonary symptoms would occur “off and on” over the course of three months. She then discovered ulcerating lesions on the proximal knuckle of her right index finger (Figure 7) and on her abdomen. Soon after, lesions appeared on her left intranasal passageway and middle of the upper lip (Figure 8). Her primary care physician treated her for squamous cell carcinoma. She presented to the hospital after the condition had not improved. Her chest x-ray exhibited pulmonary masses. Further bronchoscopy exhibited several nodules on the lungs bilaterally. She underwent a biopsy, which was conclusive for blastomycosis, and debridement with itraconazole treatment was started for six to 12 months. At her four-month follow-up visit, the patient’s cutaneous and pulmonary manifestations had resolved. Unfortunately, her post-treatment pictures are not available. 

## Discussion

These two patients both presented with cases of cutaneous, intranasal blastomycosis that enveloped both the nose and upper lip. Each of these patients' condition was preceded by pulmonary manifestations of blastomycosis. We were unable to determine whether the patients had cutaneous trauma, but they had not had any recent travel outside of West Virginia. Both patients had a history of diabetes mellitus, and given their intranasal presentation of infection, one may consider Rhizopus in the differential diagnosis. However, the patients did not present with the usual signs of facial pain, swelling, or black discharge from the sinuses. In addition, biopsy and serologic testing proved a definitive* *diagnosis of blastomycosis. Cases of blastomycosis are sporadically seen in West Virginia, and the presentation of blastomycosis of the head and neck is considered uncommon. One commonality shared by both patients is that they were given a diagnosis of malignant neoplasm, such as melanoma and squamous cell carcinoma. The consideration for a fungal infection was not assessed until the patients were transferred to a tertiary medical center. At this point, the patients underwent biopsy, which showed conclusive evidence of cutaneous blastomycosis infection. These patients were treated with debridement and itraconazole, which alleviated the patients' symptoms. This case report was designed to showcase that blastomycosis infection can be easily overlooked and mistaken for malignant neoplasms of the skin. It is important for clinicians to entertain the possibility of blastomycosis infections, especially if patients present in the southeastern states or along the Ohio and Mississippi River Valleys and Great Lakes. While cutaneous blastomycosis of the head, neck, and intranasal areas are uncommon, two cases of this condition were observed in the same region of the United States, Southern West Virginia.

## Conclusions

In this case report, we focused on the presentation of a rare cutaneous blastomycosis infection. It is interesting that two patients presented with similar rare manifestations of the disease, preceded by pulmonary complications, within the same region of the same state. There have only been four reported cases of intranasal blastomycosis as of 2016, which is increasingly of interest considering these two presented in the same region. Clinicians should be aware of the presentation of blastomycosis and consider an evaluation of the infection if patients present with ulcerating masses of head and neck, more specifically in the intranasal region. Rapid diagnosis of blastomycosis could save patients from arduous medical costs and procedures. Debridement of the masses supplemented with itraconazole will likely relieve symptoms and prevent progression of the disease.
